# *Schistosoma mansoni*‐specific immune responses and allergy in Uganda

**DOI:** 10.1111/pim.12506

**Published:** 2017-12-15

**Authors:** G. Nkurunungi, J. Kabagenyi, M. Nampijja, R. E. Sanya, B. Walusimbi, J. Nassuuna, E. L. Webb, A. M. Elliott, the LaVIISWA study team, Harriet Mpairwe, Geraldine O’Hara, Barbara Nerima, Dennison Kizito, John Vianney Tushabe, Jaco Verweij, Stephen Cose, Linda Wammes, Prossy Kabuubi, Emmanuel Niwagaba, Gloria Oduru, Grace Kabami, Elson Abayo, Eric Ssebagala, Fred Muwonge, Remy Hoek Spaans, Lawrence Muhangi, Lawrence Lubyayi, Helen Akurut, Fatuma Nalukenge, Beatrice Mirembe, Justin Okello, Sebastian Owilla, Jonathan Levin, Stephen Nash, Christopher Zziwa, Esther Nakazibwe, Josephine Tumusiime, Caroline Ninsiima, Susan Amongi, Grace Kamukama, Susan Iwala, Mirriam Akello, Robert Kizindo, Moses Sewankambo, Denis Nsubuga, Edward Tumwesige, David Abiriga, Richard Walusimbi, Victoria Nannozi, Cynthia Kabonesa, James Kaweesa, Edridah Tukahebwa, Moses Kizza, Alison Elliott

**Affiliations:** ^1^ Immunomodulation and Vaccines Programme MRC/UVRI Uganda Research Unit Entebbe Uganda; ^2^ Department of Clinical research London School of Hygiene and Tropical Medicine London UK; ^3^ College of Health Sciences Makerere University Kampala Uganda; ^4^ Department of Infectious Disease Epidemiology London School of Hygiene and Tropical Medicine London UK

**Keywords:** allergy, cytokine, ELISA, immunoglobulin, *Schistosoma* spp

## Abstract

Low allergy‐related disease (ARD) prevalence in low‐income countries may be partly attributed to helminth infections. In the *Schistosoma mansoni* (*Sm*)‐endemic Lake Victoria islands (Uganda), we recently observed positive helminth‐allergy associations, despite low ARD prevalence. To understand how *Sm*‐induced cytokine and antibody profiles might influence allergic response profiles in this population, we assessed *Schistosoma* worm (SWA)‐ and egg antigen (SEA)*‐*specific Th1 (IFN‐γ), Th2 (IL‐5, IL‐13) and regulatory (IL‐10) cytokine profiles (n = 407), and total (n = 471), SWA‐, SEA‐ and allergen (house dust mite [HDM] and cockroach)‐specific (as)IgE and IgG4 profiles (n = 2117) by ELISA. Wheeze was inversely associated with SWA‐specific IFN‐γ (*P* < .001) and IL‐10 (*P* = .058), and SEA‐specific IL‐5 (*P* = .004). Conversely, having a detectable asIgE response was positively associated with SWA‐specific IL‐5 (*P* = .006) and IL‐10 (*P* < .001). Total, SWA‐, SEA‐ and allergen‐specific IgE and IgG4 responses were higher among *Sm* Kato‐Katz positive (SmKK+) and skin prick test (SPT)+ individuals compared to SmKK‐ and SPT‐ individuals. However, total and asIgG4/IgE ratios were lower among SPT+ and wheezing individuals. We conclude that, in this population, helminth‐induced antibody and cytokine responses may underlie individual positive helminth‐atopy associations, while the overall IgG4‐IgE balance may contribute to the low overall prevalence of clinical allergies in such settings.

## 1 INTRODUCTION

Helminths have a small range of antigens that are strikingly homologous to common allergens.^1^ These antigens induce immunoglobulin (Ig) E‐mediated effector responses important for protection against helminth infection.^2^, ^3^ To survive in the host, helminths modulate this atopic pathway, and this may have a bystander protective effect against allergy‐related disease (ARD).^4^ While several animal and human studies provide compelling evidence of this protection,^5^, ^6^ others suggest that in some circumstances helminths may actually promote enhanced responses to allergens.^7^, ^8^

**Figure 1:**
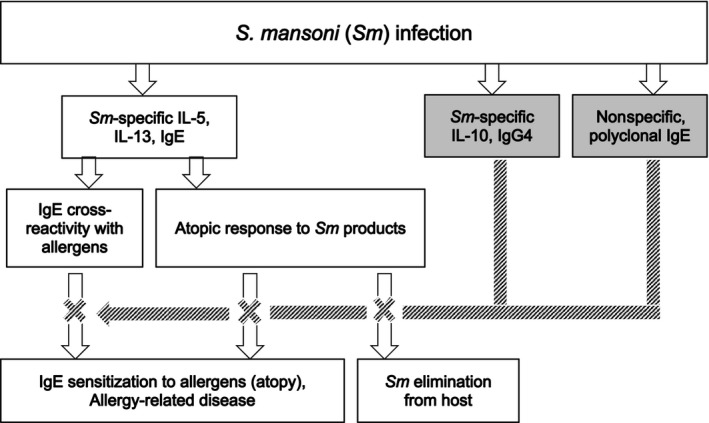
We hypothesize that the Th2 cytokine‐induced *Sm*‐specific IgE promotes potent, *Sm*‐specific, atopic effector responses and *Sm* elimination from the host, and also cross‐reactive responses to some allergens, resulting in positive *Sm*‐allergy associations. By contrast, *Sm‐*specific IL‐10, IgG4 and/or nonspecific polyclonally stimulated IgE inhibit these allergy‐related outcomes. White and shaded arrows denote promotion and inhibition, respectively

Mechanisms underlying helminth‐allergy associations in low‐income countries (LICs) are not fully understood. Hypothesized pathways that underpin these associations are shown in Figure 1. Helminth‐induced cytokine and antibody profiles may influence allergic responses and consequently epidemiological trends pertaining to ARDs.^5^, ^9^ Both helminth‐ and allergen‐specific immune responses are characterized by elevated Th2‐type responses (interleukin [IL]‐4, IL‐5 and IL‐13).^10^, ^11^ Helminths, unlike allergens, further induce strong immunoregulation epitomized by IL‐10 production.^12^ Typically, these cytokines influence the profile of antibodies involved in helminth infection and allergy. Helminth‐induced IL‐10 may drive immunoglobulin class switching to IgG4^13^, ^14^ which, akin to the Th2 cytokine‐induced^15^ polyclonally stimulated IgE, may inhibit development of allergen‐specific effector responses,^5^, ^16^ leading to inverse helminth‐allergy associations. Conversely, helminth‐induced protein‐specific IgE may promote strong, cross‐reactive helminth‐ and allergen‐specific responses, resulting in positive helminth‐allergy associations.^17^, ^18^

Emerging epidemiological data on helminth‐allergy associations in Uganda reflect the complex interaction between helminths and allergens: while observational analyses in a birth cohort suggested a protective effect of childhood and maternal helminths against childhood eczema^19^ that was reversed by maternal anthelminthic treatment,^20^ we recently reported positive helminth‐allergy associations in a survey conducted in the *Schistosoma mansoni* (*Sm*)‐endemic Lake Victoria islands, albeit against a backdrop of low ARD prevalence.^21^ To establish how *Sm‐*induced cytokine and antibody profiles underpinned helminth‐allergy associations in the above survey, we here describe an assessment of *Sm‐*specific cytokine profiles, as well as total, allergen‐ and *Sm‐*specific IgE and IgG4 profiles, and their relationship with *Sm* infection status, wheeze and atopy.

## 2 METHODS

### 2.1 Study population

Samples were collected during the baseline household survey preceding a cluster‐randomized trial of standard vs intensive anthelminthic intervention (the Lake Victoria Island Intervention Study on Worms and Allergy‐related diseases, LaVIISWA; ISRCTN47196031) described elsewhere.^21^, ^22^ Briefly, each consenting LaVIISWA participant completed a questionnaire, provided blood, urine and stool and underwent skin prick testing (SPT). Primary allergy‐related outcomes were reported wheeze in the previous 12 months and atopy. Wheeze is widely used as a surrogate for asthma in epidemiological studies^23^ and was assessed for all ages using the International Study of Asthma and Allergies in Childhood (ISAAC) questionnaire. Such symptom questionnaires have been identified as the best way to estimate asthma prevalence in epidemiological studies.^23^ The ISAAC questionnaire was used to ask participants (or their caregivers) if they had ever wheezed and if so, if they had wheezed in the last 12 months. Details on aetiology were not collected. Atopy was defined as (i) SPT reactivity to any of *Dermatophagoides* mix, *Blomia tropicalis* or German cockroach (*Blattella germanica*) [ALK‐Abelló; supplied by Laboratory Specialities Ltd., South Africa], and (ii) detectable IgE response (>312 ng/mL by ELISA^21^) to *Dermatophagoides pteronyssinus* [hereinafter “house dust mite (HDM)”] and/or German cockroach whole allergen extracts (Greer Labs, USA).

Ethics committees of Uganda Virus Research Institute, London School of Hygiene and Tropical Medicine and Uganda National Council for Science and Technology approved the study.

### 2.2 Laboratory methods

Two slides from one stool sample per individual were independently examined by different technicians for *Sm* eggs using the Kato‐Katz method.^24^

We assessed IFN‐γ (Th1‐type), IL‐5, IL‐13 (Th2‐type) and IL‐10 (regulatory) levels by ELISA using supernatants from six‐day whole blood cultures stimulated with *Schistosoma* worm (SWA) and egg antigens (SEA), as previously described.^25^ Briefly, heparinized blood was diluted with RPMI 1640 medium (Life technologies, UK) supplemented with penicillin, streptomycin, glutamine and Hepes buffer (all from Life technologies, UK), plated in 96‐well culture plates and stimulated (at 37°C, 5% CO_2_) with 10 μg/mL SWA or SEA (provided by Professor Mike Doenhoff, University of Nottingham) or mitogen (phytohaemagglutinin, PHA, Sigma, UK), or left unstimulated. Supernatants were harvested on day six and stored at −80°C until analysis. Cytokine levels in supernatants were measured by ELISA (Becton Dickinson, USA). The net response to each stimulus was calculated by subtracting the concentration in the unstimulated control well. Response values that were below the dynamic range of the assay and those that were negative after subtraction of the response in the unstimulated well were assigned a value of zero.

HDM and cockroach extract‐specific IgE and IgG4 were measured in plasma using an in‐house ELISA described previously.^20^ Briefly, MICROLON^®^ 96‐well plates (Greiner bio‐one, UK) were coated overnight at 4°C with 5 μg/mL HDM or cockroach allergens and twofold dilutions of human IgE (Calbiochem, Beeston, UK) or IgG4 (Sigma‐Aldrich) standards. Plates were blocked at room temperature (RT) with 1% skimmed milk and incubated overnight at 4°C with plasma samples diluted 1/20 (IgE assay) or 1/40 (IgG4 assay) with 10% foetal bovine serum in PBS‐Tween 20. Specific IgE or IgG4 was detected using biotinylated monoclonal mouse anti‐human IgE or IgG4 (BD Pharmingen^™^) and a streptavidin‐horseradish peroxidase conjugate (Mast Group Ltd, Bootle, UK). O‐phenylenediamine (Sigma‐Aldrich) was used as a substrate, and the reaction stopped with 2M sulphuric acid. Optical density values were measured at 490nm (reference wavelength 630nm) on a 96‐well plate ELISA reader. IgE or IgG4 concentrations (ng/mL) were interpolated from standard curves using a five‐parameter curve fit using Gen5 data collection and analysis software (BioTek Instruments Inc, Vermont, Winooski, USA). Total, SWA‐ and SEA‐specific IgE and IgG4 ELISAs were performed using similar in‐house procedures (detailed in this article's supporting information).

### 2.3 Statistical methods

Our hypothesized mode of action of *S. mansoni*‐induced cytokines and antibodies on allergy‐related outcomes is illustrated in Figure 1. Using STATA 13.1 (College Station, Texas, USA), we performed cross‐sectional analyses to assess whether *Sm* Kato‐Katz positivity and allergy‐related outcomes were associated with antibody and cytokine levels, using the “svy” command to allow for the non–self‐weighting cluster survey design. Raw cytokine and antibody responses were skewed, so log_10_ (concentration+1)‐transformed antibody and cytokine data were used in our regression models; we back‐transformed the results to obtain geometric mean ratios and 95% confidence intervals. Crude and age‐ and sex‐adjusted analyses were performed. Associations between antibody responses were estimated using Spearman's correlation coefficient (*r*_s_). We used a 5% significance level for all analyses. *P* values quoted in the main text are from adjusted analyses.

## 3 RESULTS

Questionnaire data were obtained from 2316 participants.^22^ Their characteristics and those of participants for whom cytokine and antibody responses were assessed are shown in Table 1. Participants for whom cytokine (n = 407) and total antibody levels (n = 471) were assessed were a subset of participants who had allergen‐, SWA‐ and SEA‐specific antibody results (n = 2117). Cytokine responses were assessed using samples from 1‐ to 17‐year‐olds, to allow comparison with related cellular immunology studies in an urban birth cohort (data not shown). Allergen‐, SWA‐ and SEA‐specific responses were assessed in all survey participants that had sufficient plasma sample stored.

**Table 1:** Characteristics of participants

Characteristic	Survey population (N = 2316), n/N (%)	Immunological measure, n/N (%)		
		Cytokine responses^a^(N = 407)	Allergen‐, SWA‐ and SEA‐specific IgE and IgG4^b^ (N = 2117)	Total IgE and IgG4^c^(N=471)
Age in years, median (IQR)	24 (8, 32)	9 (6, 16)	25 (10, 33)	19.5 (3, 31.25)
Male sex	1268/2316 (54.7)	168/407 (41.3)	1152/2117 (54.4)	225/471 (47.7)
PZQ in last 12 mo	382/2255 (16.9)	48/393 (12.2)	368/2062 (17.8)	15/459 (15.5)
Helminth infections				
*S. mansoni* (KK)	1041/1996 (51.4)	204/373 (54.7)	1008/1882 (53.6)	184/428 (42.9)
*S. mansoni* (urine CCA)	661/917 (72.0)	94/128 (73.4)	634/875 (72.5)	101/152 (66.5)
*S. mansoni* intensity (KK)				
Uninfected	995/1996 (48.6)	169/373 (45.3)	874/1882 (46.4)	244/428 (57.0)
Low	429/1996 (21.0)	77/373 (20.6)	411/1882 (21.8)	70/428 (16.4)
Moderate	288/1996 (13.7)	56/373 (15.0)	279/1882 (14.8)	51/428 (11.9)
Heavy	324/1996 (16.6)	71/373 (19.0)	318/1882 (16.9)	63/428 (14.7)
Any nematode infection^d^	788/2004 (39.3)	129/373 (34.6)	738/1889 (39.1)	87/428 (20.3)
Allergy‐related outcomes				
Wheeze in last 12 mo	107/2301 (4.7)	14/404 (3.5)	106/2103 (5.04)	58/468 (12.4)
Atopy (SPT)				
Any	404/1976 (19.1)	78/372 (20.9)	403/1961 (20.6)	135/448 (30.1)
*Dermatophagoides*	190/1978 (9.0)	33/372 (8.9)	189/1963 (9.6)	61/448 (13.6)
*Blomia*	205/1976 (9.6)	31/372 (8.3)	204/1961 (10.4)	67/447 (14.9)
Cockroach	272/1977 (13.2)	61/372 (16.4)	272/1962 (13.9)	90/448 (20.1)
Atopy (detectable asIgE)				
Any	1685/2117 (79.6)	320/403 (79.4)	1685/2117 (79.6)	358/471 (76.0)
*Dermatophagoides*	1534/2115 (72.5)	278/403 (68.9)	1534/2115 (72.5)	326/471 (69.2)
Cockroach	886/2117 (41.9	183/403 (45.4)	886/2117 (41.9)	186/471 (39.5)

PZQ, Praziquantel treatment; KK, Kato‐Katz; CCA, circulating cathodic antigen; SPT, skin prick test; SWA, *Schistosoma* worm antigen; SEA, *Schistosoma* egg antigen; asIgE: allergen‐specific IgE.

a Assessed using samples from 1‐ to 17‐year‐olds, to allow comparison with related cellular immunology studies in an urban birth cohort (data not shown here).

b Assessed in all survey participants that had sufficient plasma sample stored.

c Samples randomly selected from individuals with antigen‐specific antibody data.

d Infection with any of *Ascaris lumbricoides, Trichuris trichiura (assessed by KK), Necator americanus, Strongyloides stercoralis (assessed by PCR)* and *Mansonella perstans (assessed by modified Knott's method)*.

### 3.1 *S. mansoni‐*specific cytokines and allergy‐related outcomes

Individuals who tested positive for *Sm* by Kato‐Katz (SmKK+) had higher geometric mean concentrations of SWA‐specific type 2 and regulatory cytokines compared to SmKK‐ individuals (Table**** 2), but this was statistically significant only for IL‐5 (*P* = .034). However, there was no dose‐response relationship with infection intensity (Table S3A). SEA‐specific responses were similar between SmKK+ and SmKK‐ individuals.

**Table 2:** Associations between *S. mansoni*‐specific cytokine levels and (i) *S. mansoni* infection status, (ii) reported wheeze and (iii) atopy (SPT reactivity and detectable allergen‐specific IgE)

		Geometric mean^a^					
				Unadjusted		Adjusted for age and sex	
Antigen	Cytokine	SmKK‐ n = 169	SmKK+ n = 204	GMR (95% CI)^b^	*P* value	GMR (95% CI)^b^	*P* value
SWA	IFN‐γ	1.16	1.13	1.06 (0.86, 1.30)	.542	1.05 (0.87, 1.28)	.531
	IL‐5	**14.92**	**49.47**	**1.43 (1.13, 1.81)**	**.005**	**1.32 (1.02, 1.71)**	**.034**
	IL‐13	7.01	17.56	1.20 (0.94, 1.54)	.132	1.15 (0.88, 1.48)	.282
	IL‐10	3.99	11.58	1.21 (0.97, 1.51)	.084	1.16 (0.91, 1.48)	.207
SEA	IFN‐γ	0.73	0.56	0.97 (0.81, 1.17)	.760	0.98 (0.82, 1.18)	.884
	IL‐5	5.02	3.11	0.84 (0.59, 1.19)	.320	0.84 (0.58, 1.19)	.319
	IL‐13	2.25	1.95	0.86 (0.71, 1.05)	.127	0.88 (0.73, 1.06)	.190
	IL‐10	3.19	4.42	0.93 (0.78, 1.13)	.486	0.93 (0.76, 1.13)	.477

		No wheeze n = 390	Wheeze n = 14				
SWA	IFN‐γ	**1.27**	**0.23**	**0.60 (0.45, 0.80)**	**.001**	**0.57 (0.44, 0.76)**	**<.001**
	IL‐5	29.12	27.59	1.29 (0.72, 2.33)	.373	1.14 (0.63, 2.08)	.657
	IL‐13	11.75	9.78	1.26 (0.65, 2.45)	.465	1.17 (0.58, 2.36)	.635
	IL‐10	**7.91**	**2.03**	**0.69 (0.47, 1.01)**	**.059**	**0.66 (0.43, 1.02)**	**.058**
SEA	IFN‐γ	0.66	0.33	0.83 (0.66, 1.04)	.101	0.83 (0.65, 1.05)	.121
	IL‐5	**4.37**	**1.20**	**0.52 (0.33, 0.83)**	**.007**	**0.51 (0.33, 0.79)**	**.004**
	IL‐13	2.25	0.96	0.75 (0.44, 1.25)	.256	0.76 (0.44, 1.33)	.327
	IL‐10	4.34	0.59	0.70 (0.35, 1.38)	.295	0.71 (0.37, 1.36)	.291

		SPT− n = 294	SPT+^c^n = 78				
SWA	IFN‐γ	1.03	1.73	1.15 (0.96, 1.36)	.115	1.13 (0.94, 1.34)	.178
	IL‐5	29.23	39.73	1.15 (0.86, 1.52)	.330	1.02 (0.75, 1.38)	.897
	IL‐13	13.54	10.32	0.99 (0.72, 1.36)	.961	0.92 (0.67, 1.26)	.596
	IL‐10	**6.79**	**12.00**	**1.25 (1.00, 1.55)**	**.048**	1.21 (0.95, 1.54)	.126
SEA	IFN‐γ	0.55	0.99	1.11 (0.82, 1.51)	.493	1.13 (0.83, 1.52)	.423
	IL‐5	4.54	3.68	1.00 (0.70, 1.45)	.965	0.98 (0.67, 1.43)	.935
	IL‐13	2.81	1.09	0.84 (0.56, 1.25)	.376	0.85 (0.58, 1.25)	.413
	IL‐10	4.12	3.09	0.94 (0.76, 1.16)	.560	0.96 (0.75, 1.22)	.730

		Undetectable asIgE n = 83	Detectable asIgE^d^n = 320				
SWA	IFN‐γ	1.73	1.11	0.87 (0.62, 1.24)	.444	0.86 (0.60, 1.23)	.396
	IL‐5	**16.44**	**34.25**	**1.43 (1.16, 1.75)**	**.001**	**1.32 (1.09, 1.61)**	**.006**
	IL‐13	9.75	12.66	1.09 (0.77, 1.55)	.599	1.04 (0.74, 1.47)	.806
	IL‐10	**3.76**	**9.26**	**1.34 (1.18, 1.51)**	**<.001**	**1.30 (1.16, 1.46)**	**<.001**
SEA	IFN‐γ	1.12	0.56	0.79 (0.56, 1.13)	.190	0.79 (0.57, 1.11)	.176
	IL‐5	3.78	4.17	1.13 (0.81, 1.58)	.459	1.12 (0.82, 1.54)	.450
	IL‐13	2.21	2.21	0.88 (0.65, 1.22)	.449	0.90 (0.67, 1.21)	.484
	IL‐10	3.49	4.17	1.05 (0.92, 1.21)	.424	1.06 (0.93, 1.21)	.382

SmKK−, Kato‐Katz negative result (*S. mansoni*), single stool sample; SmKK+, Kato‐Katz positivity for *S. mansoni*, single stool sample; SPT, skin prick test; SWA, *Schistosoma* worm antigen; SEA, *Schistosoma* egg antigen; asIgE, allergen‐specific IgE; GMR, geometric mean ratio; 95% CI, 95% confidence interval. *P* values ≤.05 are highlighted in bold.

a All cytokine concentrations in pg/mL.

b Geometric mean ratios and 95% confidence intervals adjusted for the survey design.

c SPT reactivity to any one of *Dermatophagoides* mix, *Blomia tropicalis* or *Blattella germanica*.

d Detectable IgE to either *Dermatophagoides pteronyssinus* or *Blattella germanica*.

Wheeze was inversely associated with SWA‐specific IFN‐γ (*P* < .001) and IL‐10 (*P* = .058; Table**** 2), and SWA‐specific IL‐10/IL‐5 and SEA‐specific IL‐10/IFN‐γ ratios (Table S1). Wheezing individuals also had lower mean SEA‐specific cytokine responses, although statistical evidence for an inverse association was observed only for SEA‐specific IL‐5 (*P* = .004).

Conversely, there was a crude positive association between SPT positivity and SWA‐specific IL‐10 (*P* = .048; Table 2); and individuals with detectable allergen‐specific (as)IgE had higher SWA‐specific IL‐5 (*P* = .006) and IL‐10 responses (*P* < .001; Table**** 2) and higher SWA‐ and SEA‐specific IL‐10/IFN‐γ and IL‐5/IFN‐γ ratios (Table S1).

### 3.2 Antibody responses, *S. mansoni* Kato‐Katz positivity and allergy

Kato‐Katz positivity was strongly positively associated with SWA‐ and SEA‐specific IgE and IgG4 (*P* < .001), with HDM*‐*specific IgE (*P* = .006) and HDM*‐* and cockroach‐specific IgG4 (*P* < .001; Table**** 3 and supplementary Figure S1). However, correlations between *Sm* antigen‐specific antibodies and allergen‐specific antibodies were weak (*r*_s_ < .4, Table S2)**.**

**Table 3:** Associations between antibody (IgE and IgG4) levels and Kato‐Katz positivity (*S. mansoni*), SPT reactivity and reported wheeze

		Geometric mean^a^			
Antigen	Antibody/antibody ratio	*SmKK−*	*SmKK+*	aGMR (95% CI)^b^, ^c^	*P* value
SWA	IgE	**1080**	**2433**	**1.54 (1.28, 1.84)**	**<.001**
	IgG4	**4031**	**27 355**	**3.71 (3.14, 4.37)**	**<.001**
SEA	IgE	**1412**	**1833**	**1.32 (1.15, 1.52)**	**<.001**
	IgG4	**18 962**	**241 763**	**5.51 (4.55, 6.67)**	**<.001**
House dust mite	IgE	**0.782**	**10.678**	**1.25 (1.07, 1.45)**	**.006**
	IgG4	**0.001**	**0.192**	**1.79 (1.51, 2.13)**	**<.001**
	IgG4/IgE ratio	0.002	0.033	1.18 (0.58, 2.41)	.629
Cockroach	IgE	18.8	19.2	1.00 (0.82, 1.22)	.989
	IgG4	**0.002**	**0.292**	**1.50 (1.34, 1.68)**	**<.001**
	IgG4/IgE ratio	0.001	0.027	1.32 (0.94, 1.85)	.110
	Total IgE	**969**	**3073**	**1.37 (1.22, 1.54)**	**<.001**
	Total IgG4	**51 453**	**233 745**	**1.94 (1.49, 2.52)**	**<.001**
	Total IgG4/total IgE ratio	**52.16**	**75.24**	**1.36 (1.11, 1.67)**	**.005**
	Total IgE/cockroach IgE ratio	**3.79**	**12.60**	**1.32 (1.06, 1.66)**	**.014**
	Total IgE/dust mite IgE ratio	**0.562**	**1.301**	**1.13 (1.02, 1.25)**	**.016**

		*Cockroach SPT−*	*Cockroach SPT+*		
SWA	IgE	1704	1894	1.12 (0.94, 1.32)	.173
	IgG4	12 860	14 155	1.04 (0.85, 1.28)	.675
SEA	IgE	1611	1876	1.12 (0.97, 1.29)	.092
	IgG4	84 831	101 778	1.08 (0.92, 1.27)	.319
Dust mite	IgE	**2.6**	**42.2**	**1.59 (1.35, 1.89)**	**<.001**
	IgG4	0.022	0.061	1.06 (0.87, 1.29)	.498
	IgG4/IgE ratio	**0.009**	**0.001**	**0.56 (0.36, 0.85)**	**.010**
Cockroach	IgE	**18.9**	**39.1**	**1.25 (1.08, 1.46)**	**.004**
	IgG4	**0.054**	**0.686**	1.15 (0.95, 1.39)	.129
	IgG4/IgE ratio	0.003	0.017	0.69 (0.47, 1.02)	.064
	Total IgE	**1462**	**2787**	**1.22 (1.05, 1.42)**	**.011**
	Total IgG4	90 643	126 688	0.84 (0.65, 1.07)	.163
	Total IgG4/total IgE ratio	**60.41**	**45.80**	**0.75 (0.58, 0.95)**	**.022**
	Total IgE/cockroach IgE ratio	6.07	9.22	1.01 (0.82, 1.25)	.894
	Total IgE/dust mite IgE ratio	0.849	0.868	0.93 (0.84, 1.03)	.140

		*Dust mite SPT−*	*Dust mite SPT+*		
SWA	IgE	**1667**	**2409**	**1.26 (1.00, 1.57)**	**.043**
	IgG4	13 088	12 565	1.04 (0.79, 1.36)	.744
SEA	IgE	**1623**	**1887**	**1.23 (0.99, 1.53)**	**.055**
	IgG4	85 471	102 026	1.20 (0.85, 1.68)	.271
Dust mite	IgE	**2.5**	**242.9**	**2.10 (1.57, 2.81)**	**<.001**
	IgG4	**0.020**	**0.245**	**1.41 (0.99, 1.99)**	**.052**
	IgG4/IgE ratio	0.009	0.001	0.49 (0.23, 1.04)	.064
Cockroach	IgE	**19.9**	**33.7**	**1.21 (1.03, 1.43)**	**.024**
	IgG4	0.065	0.405	1.13 (0.85, 1.50)	.395
	IgG4/IgE ratio	0.004	0.013	0.71 (0.48, 1.04)	.075
	Total IgE	**1533**	**2802**	**1.24 (1.03, 1.49)**	**.025**
	Total IgG4	89 885	156 204	1.09 (0.86, 1.38)	.438
	Total IgG4/total IgE ratio	57.43	55.45	0.89 (0.72, 1.11)	.292
	Total IgE/cockroach IgE ratio	6.41	8.11	1.01 (0.82, 1.26)	.893
	Total IgE/dust mite IgE ratio	**0.937**	**0.517**	**0.81 (0.74, 0.89)**	**<.001**

		*No wheeze*	*Wheeze*		
SWA	IgE	1672	2627	1.15 (0.81, 1.64)	.425
	IgG4	12 753	14 802	1.05 (0.71, 1.54)	.808
SEA	IgE	1636	1547	0.93 (0.66, 1.29)	.662
	IgG4	81 698	145 978	1.16 (0.77, 1.73)	.449
House dust mite	IgE	2.8	26.6	1.35 (0.89, 2.04)	.148
	IgG4	0.007	0.008	0.85 (0.59, 1.25)	.407
	IgG4/IgE ratio	**0.011**	**0.001**	**0.45 (0.22, 0.93)**	**.032**
Cockroach	IgE	**21.2**	**2.9**	**0.69 (0.55, 0.87)**	**.003**
	IgG4	**0.021**	**0.006**	**0.65 (0.53, 0.82)**	**.001**
	IgG4/IgE ratio	0.004	0.002	1.14 (0.47, 2.74)	.760
	Total IgE	1630	1522	0.88 (0.72, 1.07)	.205
	Total IgG4	100 983	74 475	0.85 (0.61, 1.17)	.302
	Total IgG4/total IgE ratio	61.00	47.36	0.95 (0.77, 1.18)	.683
	Total IgE/cockroach IgE ratio	6.29	6.86	0.91 (0.78, 1.05)	.187
	Total IgE/dust mite IgE ratio	0.857	0.752	0.91 (0.77, 1.05)	.188

SWA, *Schistosoma* worm antigen; SEA, *Schistosoma* egg antigen; SmKK*−*, Kato‐Katz negative result (*S. mansoni*), single stool sample; SmKK+, Kato‐Katz positive result, single stool sample; aGMR, adjusted geometric mean ratio; 95% CI, 95% confidence interval. *P* values ≤0.05 are highlighted in bold.

a All antibody concentrations in ng/mL.

b All geometric mean ratios and 95% confidence intervals adjusted for survey design, age and sex.

c Geometric mean ratios and 95% confidence intervals for associations between antibody levels and SPT reactivity and wheeze were additionally adjusted for SmKK result.

Kato‐Katz positivity was also strongly positively associated with total IgE (*P* < .001) (Table 3 and Figure S1), which was in turn weakly correlated with SEA‐specific IgE but moderately correlated with SWA‐specific IgE (*r*_s_ = .31 and *r*_s_ = .51, respectively; Table S2). Similarly, total IgG4 (*P* < .001), total IgG4/total IgE ratios (*P* = .005) and total IgE/asIgE ratios (*P* < .05) were positively associated with Kato‐Katz positivity. In addition, there was a general dose‐response relationship between *S. mansoni* infection intensity and antibody responses (Table S3B).

Cockroach‐specific IgE and total IgE were positively associated with cockroach SPT reactivity. HDM‐specific IgE and IgG4, SWA‐ and SEA‐specific IgE and total IgE, were all positively associated with HDM SPT reactivity (Table 3 and Figure S1 **)**. In contrast, cockroach SPT reactivity was inversely associated with total IgG4/total IgE ratios (*P* = .022), and HDM SPT reactivity with total IgE/HDM‐specific IgE ratios (*P* < .001).

Associations between wheeze and antibody responses (Table 3 and Figure S1), when significant, were inverse. HDM IgG4/IgE ratios (*P* = .032), cockroach‐specific IgE (*P* = .003) and cockroach‐specific IgG4 (*P* = .001) were all inversely associated with wheeze.

## 4 DISCUSSION

In this highly *Sm*‐endemic setting, associations between wheeze and *Sm*‐specific cytokines and antibodies, when significant, were inverse. However, SPT reactivity and detectable asIgE were positively associated with the same *Sm*‐specific responses.

In this population, *Sm* exposure is almost universal, and infection much higher than indicated by Kato‐Katz: urine assessment for *Sm* circulating cathodic antigen (CCA) indicated a prevalence of over 70%, compared to 51.4% prevalence by Kato‐Katz.^22^ Therefore, Kato‐Katz negativity in many study participants was indicative of lighter (rather than absent) infection. This explains why, although SWA‐specific Th2‐type and regulatory cytokine responses were generally higher among SmKK+ individuals, only SWA‐specific IL‐5 reached significant levels, and why SEA‐specific responses were similar between SmKK+ and SmKK‐ individuals. Further support for these observations comes from supplementary analysis (Table S4A), which shows that cytokine responses were similar between SmKK‐CCA+ and SmKK+CCA± individuals.

All statistically significant associations between atopy and *Sm*‐specific cytokine responses were positive. Associations with whole blood cytokine responses are best interpreted taking into account total cell counts, but these data were unavailable. However, atopy‐antibody associations were also positive. Besides, these results mirror our previous epidemiological observations in this population, where *Sm* infection was positively associated with *Dermatophagoides*‐specific IgE, and atopy‐wheeze associations were stronger in the presence of *Sm* infection.^21^

Our results were unexpected in view of earlier findings from Gabon^9^ which showed an inverse association between dust mite SPT and SWA‐specific IL‐10 (albeit we used whole blood cultures, compared to peripheral blood mononuclear cells in the Gabon study). However, although IL‐10 is chiefly immunomodulatory,^26^, ^27^, ^28^ it may also enhance IgE production in already IgE‐switched B cells;^13^ these may be abundant in individuals from this helminth‐endemic setting. SWA‐ and SEA‐specific IgE were weakly positively associated with HDM SPT reactivity, perhaps unsurprisingly, as helminth antigens may induce cross‐reactive helminth‐ and allergen‐specific IgE effector responses. Total serum IgE, elevated during helminth infection mainly due to increased synthesis of polyclonal IgE, has been proposed to inhibit allergic responses.^29^, ^30^ However, contrasting evidence links high serum IgE levels to increased expression of IgE receptors on human basophils,^31^ and we show positive associations between total IgE and SPT reactivity to both cockroach and dust mite.

In keeping with the original hypothesis, associations between wheeze and cytokine and antibody responses, when significant, were inverse. Furthermore, total and allergen‐specific IgG4/IgE ratios were mostly inversely associated with atopy, implying that the regulatory role of IgG4 against allergy might best be assessed relative to IgE. Also, lower total/asIgE ratios among HDM SPT+ individuals are consistent with the perception that high total/asIgE ratios may be protective against allergic responses, because nonspecific polyclonal IgE may compete with asIgE to saturate IgE receptors.^29^

One limitation of assessing helminth‐allergy associations and underlying mechanisms in this population is the almost universal exposure to helminths, and lack of data on duration of infection. We also report a large number of statistical tests, so some apparently “significant” findings could have occurred by chance. As we anticipated that some of our measures might be correlated, we did not formally adjust for multiplicity, instead we focussed on patterns of association and consistency of results, and on biological plausibility with reference to other findings. Another potential limitation is that wheeze was relatively rare in the study population, and hence, some of our comparison groups (such as the age group 1‐17 years) had a low prevalence. Besides, reported wheeze could easily be misclassified in this population due to lack of a direct translation of “wheeze” in the native languages.^21^

Nonetheless, our results generally agree with our epidemiological observations in the same population,^21^ where we found a very low prevalence of clinical allergies, despite positive helminth‐atopy associations.

## DISCLOSURES

None.

## AUTHOR CONTRIBUTIONS

AME conceived the main study. AME, RES and MN led the field and clinic teams. AME, GN and JK participated in the design of laboratory studies. GN, JK, BW and JN performed the experiments. GN and ELW analysed the results. GN wrote the manuscript, with all authors contributing to the interpretation of the results, and revision and approval of the final manuscript. GN is the guarantor of the article.

## APPENDIX 1

### LaVIISWA trial team

*Project leaders, physicians and postdoctoral scientists:* Richard Sanya, Margaret Nampijja, Harriet Mpairwe, Geraldine O'Hara, Barbara Nerima*. Laboratory staff and collaborators:* Gyaviira Nkurunungi, Joyce Kabagenyi, Dennison Kizito, John Vianney Tushabe, Jacent Nassuuna, Jaco Verweij, Stephen Cose, Linda Wammes, Prossy Kabuubi, Emmanuel Niwagaba, Gloria Oduru, Grace Kabami, Elson Abayo, Eric Ssebagala, Fred Muwonge. *Statisticians and data managers:* Emily Webb, Remy Hoek Spaans, Lawrence Muhangi, Lawrence Lubyayi, Helen Akurut, Fatuma Nalukenge, Beatrice Mirembe, Justin Okello, Sebastian Owilla, Jonathan Levin, Stephen Nash. *Clinical officers:* Milly Namutebi, Christopher Zziwa. *Nurses:* Esther Nakazibwe, Josephine Tumusiime, Caroline Ninsiima, Susan Amongi, Grace Kamukama, Susan Iwala, Florence. *Internal monitor:* Mirriam Akello. *Field workers:* Robert Kizindo, Moses Sewankambo, Denis Nsubuga. *Social sciences:* Edward Tumwesige. *Boatman:* David Abiriga. *Driver:* Richard Walusimbi. *HIV counselling and testing:* Victoria Nannozi, Cynthia Kabonesa. *Vector Control Programme staff:* James Kaweesa, Edridah Tukahebwa. *Administrative management:* Moses Kizza. *Principal investigator:* Alison Elliott.
