# Supplementary material for: Schistosoma mansoni‐specific immune responses and allergy in Uganda
Source: Parasite Immunol. 2017 Dec 15;40(1):e12506. doi: 10.1111/pim.12506 (PMC5767746; doi:10.1111/pim.12506)
Supplement: Supplementary file 1 [file PIM-40-na-s001.docx]

***Schistosoma mansoni*-specific antibody and cytokine profiles are positively associated with atopy, but not wheeze, in Lake Victoria fishing communities, Uganda**

Gyaviira Nkurunungi^1,2^*, Joyce Kabagenyi^1^, Margaret Nampijja^1^, Richard E Sanya^1,3^, Bridgious Walusimbi^1^, Jacent Nassuuna^1^, Emily L Webb^4^, Alison M Elliott^1,2^ **for the LaVIISWA study team**

^1^Immunomodulation and Vaccines Programme, MRC/UVRI Uganda Research Unit, Entebbe, Uganda

^2^Department of Clinical research, London School of Hygiene and Tropical Medicine, London, United Kingdom

^3^College of Health Sciences, Makerere University, Kampala, Uganda

^4^Department of Infectious Disease Epidemiology, London School of Hygiene and Tropical Medicine, London, United Kingdom

**Supporting information**

# Experimental methods

## S. mansoni worm (SWA)- and egg (SEA)-specific IgE and IgG4 ELISA

All but the first 2 columns of 4HX Immulon (Thermo Scientific, NY, USA) 96-well plates were coated with 50μl of SWA [8 μg/ml] or SEA [2.4 μg/ml] (purchased from Professor Mike Doenhoff, University of Nottingham) in bicarbonate buffer (0.1M, pH 9.6). Two-fold dilutions of human IgE (Calbiochem, Beeston, UK) or IgG4 (Sigma-Aldrich) standard, diluted in bicarbonate buffer, were added to the first 2 columns of each plate to form standard curves. The plates were then incubated overnight at 4^0^C. Plates were washed with phosphate-buffered saline (PBS)-tween 20 solution, blocked with 150μl of skimmed milk diluted in PBS-Tween 20 at room temperature (RT), and incubated overnight at 4^0^C with 50μl of plasma samples diluted 1/20 with 10% fetal bovine serum in PBS-Tween 20 (assay buffer). Plates were washed and antibody binding detected by incubating the plates overnight at 4^0^C with 0.5μg/ml of biotinylated monoclonal mouse anti-human IgE or IgG4 (BD Pharmingen™), followed by a 1 hour incubation with a streptavidin-Horseradish Peroxidase (strep-HRP) conjugate (Mast Group Ltd, Bootle, UK), diluted 1/3000 with assay buffer, at RT. Plates were developed by addition of 100μl of o-phenylenediamine (Sigma-Aldrich) and reactions stopped with 30μl of 2M Sulphuric acid. Optical density values were measured at 490nm (reference wavelength 630nm) on a 96-well plate ELISA reader. IgE or IgG4 concentrations (ng/ml) were interpolated from standard curves using a five-parameter curve fit using Gen5 data collection and analysis software (BioTek Instruments Inc, Vermont, Winooski, USA).

## Total IgE ELISA

96-well Maxisorp plates (VWR, U.S.A) were coated with 100μl of polyclonal rabbit anti human IgE (Agilent technologies, Dako, Denmark) diluted 1/1000 in bicarbonate buffer (0.1M, pH 9.6) and incubated overnight at 4^o^C. Plates were then washed with phosphate-buffered saline (PBS)-tween 20 solution and blocked with 120μl of PBS-bovine serum albumin (BSA) solution for 1 hour at room temperature (RT). Plasma samples (100μl) diluted 1/50 in assay buffer (0.1M Tris pH 7.5 + 0.05% Tween-20), the blank (assay buffer) and National Institute for Biological Standards and Control (NIBSC) international IgE standards were added to the plates and incubated for 1 hour at RT. Plates were then washed and incubated with 100μl of biotinylated goat anti-human IgE (Vector laboratories, U.S.A, 0.5mg/ml), diluted 1/1000 with assay buffer, for 1 hour at RT. After another washing step, the plates were incubated with 100μl of streptavidin alkaline phosphatase (Roche Life Science), diluted 1/3000 with assay buffer, for 30 minutes at RT. 4-nitrophenyl phosphate disodium salt hexahydrate (p-NPP), diluted in diethanolamine buffer (DEA, 0.1M), was added, followed by 20 minutes incubation at RT in the dark for development. Sodium hydroxide (3M, 100μl) was then added to stop the reaction. Plates were read at 405 nm using an ELISA reader. Results were interpolated from standard curves using a five-parameter curve fit using Gen5 data collection and analysis software (BioTek Instruments Inc, Vermont, Winooski, USA).

## Total IgG4 ELISA

96-well Maxisorp plates (VWR, U.S.A) were coated with purified mouse anti-human IgG4 (BD Pharmingen™) in bicarbonate buffer (0.1 M, pH 9.6) overnight at 4^o^C, and blocked for 1 hour with 3% skimmed milk in 1X PBS at room temperature (RT). Plates were then incubated with plasma samples (diluted 1/800 in 0.1M Tris pH 7.5 + 0.05% Tween-20) and IgG4 standards (Sigma Aldrich) for one hour. Antibody binding was detected by incubating the plates with mouse anti-human IgG4 conjugated to horseradish peroxidase (Invitrogen) for one hour, followed by a colour reaction with o-phenylenediamine (Sigma Aldrich). Reactions were stopped with 2M Sulphuric acid. Absorbance was measured at 490nm (reference wavelength 630nm) on a 96-well plate ELISA reader. IgG4 concentrations (ng/ml) were interpolated from standard curves using a five-parameter curve fit using Gen5 data collection and analysis software (BioTek Instruments Inc, U.S.A).

# Supplementary tables and figures

**Table S1.** Associations between *S. mansoni*-specific cytokine ratios and i) *S. mansoni* infection status, ii) reported wheeze and iii) atopy (SPT reactivity and detectable allergen-specific IgE)

|  |  |  | |  | **Unadjusted** | |  | **Adjusted for age and sex** | |
| --- | --- | --- | --- | --- | --- | --- | --- | --- | --- |
| **Antigen** | **Ratio** | **Geometric mean**^†^ | |  | **GMR (95% CI)**^‡^ | **p value** |  | **GMR (95% CI)** ^‡^ | **p value** |
|  |  | SmKK-  n=169 | SmKK+  n=204 |  |  |  |  |  |  |
| **SWA** | IL-10/ IFN-γ | 3.29 | 10.46 |  | 1.23 (0.89, 1.71) | 0.192 |  | 1.18 (0.85, 1.65) | 0.297 |
|  | IL-10/ IL-5 | 0.26 | 0.23 |  | 0.91 (0.74, 1.11) | 0.337 |  | 0.92 (0.74, 1.15) | 0.456 |
|  | IL-10/ IL-13 | 0.56 | 0.65 |  | 0.95 (0.71, 1.29) | 0.767 |  | 0.95 (0.67, 1.33) | 0.745 |
|  | IL-5/ IFN-γ | **12.79** | **43.52** |  | **1.36 (1.03, 1.80)** | **0.028** |  | 1.25 (0.94, 1.65) | 0.120 |
|  | IL-13/ IFN-γ | 6.01 | 15.45 |  | 1.11 (0.81, 1.53) | 0.498 |  | 1.03 (0.75, 1.43) | 0.824 |
| **SEA** | IL-10/ IFN-γ | 4.54 | 8.14 |  | 1.00 (0.75, 1.34) | 0.985 |  | 0.97 (0.73, 1.29) | 0.849 |
|  | IL-10/ IL-5 | 0.65 | 1.39 |  | 0.97 (0.72, 1.31) | 0.853 |  | 0.96 (0.71, 1.32) | 0.826 |
|  | IL-10/ IL-13 | 1.42 | 2.16 |  | 0.96 (0.72, 1.29) | 0.807 |  | 0.95 (0.69, 1.32) | 0.768 |
|  | IL-5/ IFN-γ | 6.88 | 5.55 |  | 0.94 (0.69, 1.29) | 0.723 |  | 0.91 (0.66, 1.25) | 0.550 |
|  | IL-13/ IFN-γ | 3.09 | 3.49 |  | 0.85 (0.67, 1.07) | 0.175 |  | 0.85 (0.68, 1.05) | 0.123 |
|  |  | No wheeze  n=390 | Wheeze  n=14 |  |  |  |  |  |  |
| **SWA** | IL-10/ IFN-γ | 6.22 | 8.82 |  | 0.99 (0.68, 1.42) | 0.961 |  | 0.97 (0.61, 1.55) | 0.902 |
|  | IL-10/ IL-5 | **0.26** | **0.07** |  | **0.79 (0.69, 0.91)** | **0.001** |  | **0.83 (0.70, 0.97)** | **0.023** |
|  | IL-10/ IL-13 | 0.66 | 0.21 |  | 0.78 (0.53, 1.15) | 0.206 |  | 0.79 (0.52, 1.21) | 0.267 |
|  | IL-5/ IFN-γ | **22.87** | **119.94** |  | **2.51 (1.14, 5.51)** | **0.024** |  | **2.25 (1.03, 4.96)** | **0.044** |
|  | IL-13/ IFN-γ | 9.23 | 42.54 |  | 2.21 (0.91, 5.43) | 0.079 |  | 2.08 (0.85, 5.06) | 0.100 |
| **SEA** | IL-10/ IFN-γ | **6.78** | **1.78** |  | **0.52 (0.34, 0.80)** | **0.004** |  | **0.53 (0.37, 0.74)** | **0.001** |
|  | IL-10/ IL-5 | 0.99 | 0.49 |  | 1.44 (0.48, 4.24) | 0.493 |  | 1.49 (0.53, 4.21) | 0.432 |
|  | IL-10/ IL-13 | 1.88 | 0.61 |  | 0.78 (0.53, 1.16) | 0.212 |  | 0.79 (0.55, 1.15) | 0.220 |
|  | IL-5/ IFN-γ | 6.59 | 3.62 |  | 0.57 (0.32, 1.05) | 0.069 |  | 0.55 (0.29, 1.02) | 0.058 |
|  | IL-13/ IFN-γ | 3.40 | 2.90 |  | 0.69 (0.46, 1.03) | 0.072 |  | 0.69 (0.45, 1.06) | 0.088 |
|  |  | SPT-  n=294 | SPT+^§^  n=78 |  |  |  |  |  |  |
| **SWA** | IL-10/ IFN-γ | 6.68 | 6.85 |  | 0.97 (0.76, 1.25) | 0.840 |  | 0.94 (0.73, 1.22) | 0.639 |
|  | IL-10/ IL-5 | 0.22 | 0.32 |  | 1.06 (0.86, 1.30) | 0.532 |  | 1.11 (0.92, 1.34) | 0.276 |
|  | IL-10/ IL-13 | 0.48 | 1.22 |  | 1.28 (0.94, 1.74) | 0.107 |  | 1.31 (0.99, 1.74) | 0.056 |
|  | IL-5/ IFN-γ | 28.51 | 22.94 |  | 0.99 (0.74, 1.33) | 0.954 |  | 0.87 (0.67, 1.14) | 0.324 |
|  | IL-13/ IFN-γ | 13.2 | 5.96 |  | 0.89 (0.62, 1.27) | 0.515 |  | 0.83 (0.59, 1.15) | 0.253 |
| **SEA** | IL-10/ IFN-γ | 7.53 | 3.37 |  | 0.88 (0.63, 1.23) | 0.444 |  | 0.88 (0.63, 1.22) | 0.432 |
|  | IL-10/ IL-5 | 0.88 | 0.92 |  | 1.01 (0.82, 1.23) | 0.955 |  | 1.05 (0.85, 1.28) | 0.635 |
|  | IL-10/ IL-13 | 1.37 | 3.15 |  | 1.26 (0.89, 1.77) | 0.175 |  | 1.31 (0.95, 1.83) | 0.095 |
|  | IL-5/ IFN-γ | 8.11 | 3.71 |  | 0.86 (0.59, 1.26) | 0.435 |  | 0.81 (0.55, 1.16) | 0.234 |
|  | IL-13/ IFN-γ | 5.01 | 1.09 |  | 0.72 (0.50, 1.02) | 0.064 |  | 0.71 (0.50, 1.00) | 0.051 |
|  |  | Undetectable asIgE  n=83 | Detectable asIgE^¶^  n=320 |  |  |  |  |  |  |
| **SWA** | IL-10/ IFN-γ | **2.05** | **8.44** |  | **1.47 (1.07, 2.02)** | **0.019** |  | **1.46 (1.09, 1.94)** | **0.012** |
|  | IL-10/ IL-5 | 0.22 | 0.26 |  | 0.99 (0.83, 1.20) | 0.999 |  | 1.02 (0.86, 1.23) | 0.749 |
|  | IL-10/ IL-13 | 0.36 | 0.72 |  | 1.09 (0.87, 1.37) | 0.430 |  | 1.10 (0.88, 1.36) | 0.370 |
|  | IL-5/ IFN-γ | **9.47** | **30.88** |  | **1.69 (1.21, 2.37)** | **0.003** |  | **1.58 (1.17, 2.14)** | **0.004** |
|  | IL-13/ IFN-γ | 5.62 | 11.42 |  | 1.31 (0.96, 1.77) | 0.080 |  | 1.25 (0.96, 1.62) | 0.093 |
| **SEA** | IL-10/ IFN-γ | **2.97** | **7.79** |  | 1.41 (0.99, 1.99) | 0.055 |  | **1.43 (1.03, 1.96)** | **0.032** |
|  | IL-10/ IL-5 | 0.88 | 1.01 |  | 0.97 (0.73, 1.30) | 0.878 |  | 0.99 (0.77, 1.28) | 0.972 |
|  | IL-10/ IL-13 | 1.51 | 1.85 |  | 1.04 (0.76, 1.41) | 0.785 |  | 1.05 (0.79, 1.39) | 0.703 |
|  | IL-5/ IFN-γ | **3.37** | **7.38** |  | **1.58 (1.16, 2.15)** | **0.005** |  | **1.55 (1.15, 2.08)** | **0.005** |
|  | IL-13/ IFN-γ | 1.96 | 3.89 |  | 1.13 (0.92, 1.40) | 0.233 |  | 1.14 (0.93, 1.39) | 0.186 |
| ^†^All antibody concentrations in pg/ml.  ^‡^Geometric mean ratios and 95% confidence intervals adjusted for the survey design.  ^§^SPT reactivity to any one of *Dermatophagoides* mix, Blomia tropicalis or *Blattella germanica.*  ^¶^Detectable IgE to either *Dermatophagoides pteronyssinus* or *Blattella germanica*.  SmKK-: Kato-Katz negative result (*S. mansoni*), single stool sample; SmKK+: Kato-Katz positivity for *S. mansoni*, single stool sample; SWA: *Schistosoma* worm antigen; SEA: *Schistosoma* egg antigen; asIgE: allergen-specific IgE; GMR: geometric mean ratio; 95% CI: 95% confidence interval. | | | | | | | | | |

**Figure S1.** Summary of associations between antibody levels and i) *S. mansoni* infection, ii) SPT reactivity and iii) wheeze

Positive association, p <0.1

Positive association, p <0.05

Positive association, p <0.01

Positive association, p <0.001

Inverse association, p <0.1

Inverse association, p <0.05

Inverse association, p <0.01

Inverse association, p <0.001

*S. mansoni* infection (Kato-Katz)

Reported wheeze

Dust mite SPT

SWA IgE

SEA IgG4

Total IgE

Dust mite IgG4

Dust mite IgE

Dust mite IgG4/IgE ratio

Cockroach IgE

Cockroach IgG4

SEA IgE

Total IgE/cockroach IgE ratio

Total IgE/dust mite IgE ratio

SWA IgG4

Total IgG4/total IgE ratio

Total IgG4

Cockroach SPT

Cockroach IgG4/IgE ratio

Red and blue arrows denote positive and inverse associations, respectively. The thickness of the arrows shows the level of statistical significance. P values were obtained from linear regression analyses, after adjusting for survey design, age and sex. Furthermore, analyses assessing associations between antibody levels and SPT reactivity and wheeze were additionally adjusted for *S. mansoni* infection (Kato-Katz). SPT: skin prick test; SWA: *Schistosoma* worm antigen; SEA: *Schistosoma* egg antigen.

**Table S2.** Correlation between antibody profiles

|  | **SWA IgE** | **SEA IgE** | **SWA IgG4** | **SEA IgG4** | **Dust mite IgE** | **Cockroach IgE** | **Dust mite IgG4** | **Cockroach IgG4** | **Total IgE** | **Total IgG4** |
| --- | --- | --- | --- | --- | --- | --- | --- | --- | --- | --- |
| **SWA IgE** | 1 |  |  |  |  |  |  |  |  |  |
| **SEA IgE** | 0.5202 | 1 |  |  |  |  |  |  |  |  |
| **SWA IgG4** | 0.4409 | 0.2622 | 1 |  |  |  |  |  |  |  |
| **SEA IgG4** | 0.3783 | 0.3074 | 0.6997 | 1 |  |  |  |  |  |  |
| **Dust mite IgE** | 0.3823 | 0.2631 | 0.2462 | 0.218 | 1 |  |  |  |  |  |
| **Cockroach IgE** | 0.2302 | 0.2895 | 0.0992 | 0.0707 | 0.3539 | 1 |  |  |  |  |
| **Dust mite IgG4** | 0.1217 | 0.1702 | 0.2756 | 0.2425 | 0.1589 | 0.013 | 1 |  |  |  |
| **Cockroach IgG4** | 0.1485 | 0.2062 | 0.2423 | 0.1701 | 0.1055 | 0.1014 | 0.5729 | 1 |  |  |
| **Total IgE** | 0.5128 | 0.3095 | 0.4796 | 0.3764 | 0.3767 | 0.1575 | 0.1708 | 0.211 | 1 |  |
| **Total IgG4** | 0.2201 | 0.2042 | 0.5052 | 0.4573 | 0.0861 | 0.0147 | 0.3902 | 0.3371 | 0.3977 | 1 |
|  | | | | | | | | | | |
| Table shows Spearman’s rank correlation coefficients (r_s_), calculated to estimate strength of association between antibody responses. Hinkle and colleagues’ criteria**^1^** were used to interpret r_s_ values: 0.7 – 1.00: high correlation; 0.5 – 0.7: moderate correlation; 0.3 – 0.5: low correlation; 0.00 – 0.3: little if any correlation. | | | | | | | | | | |

**Table S3.** Associations between *S. mansoni* infection intensity and antibody and cytokine responses

| **A** | | | | | | | | | | |
| --- | --- | --- | --- | --- | --- | --- | --- | --- | --- | --- |
| **Antigen** | **Cytokine** | **Geometric mean**^†^ | | | | |  | **aGMR (95% CI)** ^‡^ | | |
|  |  | SmKK- | | SmKK^low^ | SmKK^mod^ | SmKK^heavy^ |  | SmKK^low^ | SmKK^mod^ | SmKK^heavy^ |
| SWA | IFN-γ | 1.16 | | 2.67 | **1.14** | 0.45 |  | 1.49 (0.99, 2.25) | 0.96 (0.70, 1.33) | **0.77 (0.61, 0.96)** |
|  | IL-5 | **14.92** | | 28.67 | **80.23** | 61.04 |  | 1.09 (0.82, 1.45) | **1.57 (1.09, 2.25)** | 1.42 (0.95, 2.09) |
|  | IL-13 | 7.01 | | 15.58 | 17.63 | 19.92 |  | 1.11 (0.85, 1.45) | 1.17 (0.85, 1.60) | 1.17 (0.79, 1.72) |
|  | IL-10 | 3.99 | | 9.65 | 13.68 | 12.39 |  | 1.08 (0.79, 1.49) | 1.18 (0.87, 1.61) | 1.25 (1.03, 1.51) |
| SEA | IFN-γ | 0.73 | | 1.25 | 0.42 | **0.29** |  | 1.26 (0.96, 1.65) | 0.92 (0.68, 1.25) | **0.78 (0.67, 0.92)** |
|  | IL-5 | 5.02 | | 6.18 | 3.08 | **1.49** |  | 1.10 (0.75, 1.62) | 0.75 (0.49, 1.14) | **0.67 (0.48, 0.93)** |
|  | IL-13 | 2.25 | | 5.64 | 1.50 | **0.76** |  | 1.22 (0.98, 1.51) | 0.78 (0.61, 1.02) | **0.66 (0.54, 0.83)** |
|  | IL-10 | 3.19 | | 7.32 | 4.63 | 2.45 |  | 1.12 (0.91, 1.38) | 0.88 (0.67, 1.16) | 0.78 (0.59, 1.05) |
|  | | | | | | | | | | |
| **B** | | | | | | | | | | |
| **Antigen** | **Antibody/**  **antibody ratio** | | **Geometric mean**^¶^ | | | |  | **aGMR (95% CI)** ^‡^ | | |
|  |  | | SmKK- | SmKK^low^ | SmKK^mod^ | SmKK^heavy^ |  | SmKK^low^ | SmKK^mod^ | SmKK^heavy^ |
| SWA | IgE | | **1080** | **2075** | **2544** | **2849** |  | **1.38 (1.08, 1.75)** | **1.48 (1.21, 1.82)** | **1.84 (1.52, 2.23)** |
|  | IgG4 | | **4031** | **14132** | **26403** | **62356** |  | **2.69 (2.28, 3.16)** | **3.77 (2.82, 5.05)** | **5.75 (4.41, 7.50)** |
| SEA | IgE | | **1412** | **1870** | **1953** | **1688** |  | **1.22 (1.06, 1.39)** | **1.50 (1.26, 1.78)** | **1.35 (1.06, 1.71)** |
|  | IgG4 | | **18962** | **170274** | **229400** | **391440** |  | **4.45 (3.47, 5.71)** | **5.86 (4.66, 7.37)** | **7.04 (5.59, 8.87)** |
| Dust mite | IgE | | **0.782** | 3.926 | **10.552** | **39.482** |  | 1.05 (0.86, 1.27) | **1.33 (1.12, 1.56)** | **1.51 (1.14, 2.01)** |
|  | IgG4 | | **0.001** | **0.059** | **0.274** | **5.939** |  | **1.50 (1.18, 1.91)** | **1.65 (1.31, 2.08)** | **2.51 (2.04, 3.07)** |
|  | IgG4/IgE ratio | | 0.002 | 0.013 | 0.024 | 0.155 |  | 1.37 (0.62, 3.03) | 1.10 (0.67, 1.81) | 1.03 (0.37, 2.82) |
| Cockroach | IgE | | 18.87 | 15.60 | 16.66 | 28.37 |  | 0.97 (0.78, 1.21) | 1.02 (0.74, 1.42) | 1.02 (0.86, 1.19) |
|  | IgG4 | | **0.002** | **0.091** | **0.147** | **3.349** |  | **1.43 (1.25, 1.65)** | **1.43 (1.22, 1.68)** | **2.01 (1.69, 2.39)** |
|  | IgG4/IgE ratio | | 0.001 | 0.009 | 0.015 | 0.176 |  | 1.35 (0.91, 2.03) | 1.08 (0.64, 1.83) | 1.49 (0.91, 2.44) |
|  | | | | | | | | | | |
|  | Total IgE | **969** | | **2548** | **2208** | **4946** |  | **1.21 (1.01, 1.45)** | **1.19 (1.06, 1.35)** | **1.80 (1.62, 2.00)** |
|  | Total IgG4 | **51453** | | **115588** | **235755** | **520703** |  | **1.57 (1.31, 1.89)** | **2.07 (1.53, 2.66)** | **2.42 (1.59, 3.66)** |
|  | Total IgG4/ total IgE ratio | **52.16** | | 45 | **107** | **100** |  | 1.21 (0.96, 1.52) | **1.56 (1.17, 2.08)** | **1.39 (1.08, 1.79)** |
|  | Total IgE/ cockroach IgE ratio | **3.79** | | 11.23 | 8.47 | **19.30** |  | 1.27 (0.92, 1.76) | 1.08 (0.87, 1.36) | **1.64 (1.31, 2.04)** |
|  | Total IgE/ dust mite IgE ratio | **0.562** | | **1.66** | 0.78 | 1.52 |  | **1.17 (1.02, 1.32)** | 1.04 (0.86, 1.26) | **1.17 (1.03, 1.34)** |
| ^†^All cytokine concentrations in pg/ml.  ^¶^All antibody concentrations in ng/ml.  ^‡^Geometric mean ratios and 95% confidence intervals adjusted for the survey design, age and sex.  **SmKK-**: Kato-Katz negative result (*S. mansoni*), single stool sample; **SmKK^low^**: Kato-Katz positivity, low infection intensity (1-99 eggs/g); **SmKK^mod^**: Kato-Katz positivity, moderate infection intensity (100-399 eggs/g); **SmKK^heavy^**: Kato-Katz positivity, heavy infection intensity (≥400 eggs/g); **SWA**: *Schistosoma* worm antigen; **SEA**: *Schistosoma* egg antigen; **aGMR**: adjusted geometric mean ratio; **95% CI**: 95% confidence interval. | | | | | | | | | | |

**Table S4.** Comparison of cytokine and antibody responses between SmKK-CAA+ participants and (i) SmKK-CCA- and (ii) SmKK+CAA+/- individuals**^†^**

| **A** | | | | | | | |
| --- | --- | --- | --- | --- | --- | --- | --- |
| **Antigen** | **Cytokine** | **Geometric mean** | | |  | **aGMR (95% CI)** **^‡§^** | |
|  |  | *SmKK-CCA+* | *SmKK-CCA-* | *SmKK+CCA+/-* |  | *SmKK-CCA-* | *SmKK+CCA+/-* |
| SWA | IFN-γ | 1.83 | **0.57** | 1.33 |  | **0.71 (0.52, 0.97)** | 0.90 (0.66, 1.23) |
|  | IL-5 | 17.81 | 10.80 | 40.28 |  | 0.80 (0.47, 1.35) | 1.38 (0.95, 2.01) |
|  | IL-13 | 9.54 | 5.91 | 14.62 |  | 0.71 (0.43, 1.19) | 1.06 (0.68, 1.65) |
|  | IL-10 | 5.72 | **2.15** | 15.27 |  | **0.68 (0.55, 0.86)** | 1.25 (0.93, 1.69) |
| SEA | IFN-γ | 0.57 | 1.09 | 0.46 |  | 0.98 (0.56, 1.74) | 0.96 (0.63, 1.46) |
|  | IL-5 | 15.15 | **2.35** | 3.26 |  | **0.49 (0.33, 0.74)** | 0.69 (0.46, 1.04) |
|  | IL-13 | 2.79 | **1.15** | 1.85 |  | **0.66 (0.44, 1.00)** | 0.96 (0.56, 1.65) |
|  | IL-10 | 5.40 | **1.63** | 5.45 |  | **0.66 (0.45, 0.98)** | 0.97 (0.66, 1.41) |
| **B** | | | | | | | |
| **Antigen** | **Antibody /**  **antibody ratio** | **Geometric mean** | | |  | **aGMR (95% CI)** ^‡^ | |
|  |  | *SmKK-CCA+* | *SmKK-CCA-* | *SmKK+CCA+/-* |  | *SmKK-CCA-* | *SmKK+CCA+/-* |
| SWA | IgE | 1244 | 843 | **3058** |  | 0.92 (0.50, 1.67) | **1.67 (1.13, 2.48)** |
|  | IgG4 | 4830 | **2291** | **27587** |  | **0.60 (0.45, 0.79)** | **2.85 (2.01, 4.03)** |
| SEA | IgE | 1473 | 1453 | **1927** |  | 0.76 (0.43, 1.37) | **1.42 (1.14, 1.76)** |
|  | IgG4 | 33789 | **6311** | **218820** |  | **0.30 (0.14, 0.65)** | **3.63 (2.28, 5.77)** |
| Dust mite | IgE | 0.61 | 0.45 | **22.59** |  | 1.06 (0.82, 1.38) | **1.46 (1.16, 1.84)** |
|  | IgG4 | 0.001 | 0.001 | **0.221** |  | 1.03 (0.75, 1.39) | **1.46 (1.12, 1.92)** |
|  | IgG4/IgE ratio | 0.004 | 0.004 | 0.015 |  | 0.79 (0.32, 1.95) | 0.57 (0.27, 1.21) |
| Cockroach | IgE | 20.17 | 9.61 | 17.85 |  | 0.92 (0.74, 1.14) | 0.98 (0.75, 1.27) |
|  | IgG4 | 0.079 | 0.005 | **0.609** |  | 0.82 (0.63, 1.06) | **1.26 (1.09, 1.47)** |
|  | IgG4/IgE ratio | 0.004 | 0.000 | 0.035 |  | 0.86 (0.49, 1.51) | 1.24 (0.78, 1.96) |
|  |  |  |  |  |  |  |  |
|  | Total IgE | 696 | 1222 | **3302** |  | 1.16 (0.82, 1.65) | **1.69 (1.15, 2.48)** |
|  | Total IgG4 | 44075 | 5543 | **282711** |  | 0.91 (0.67, 1.21) | **1.79 (1.10, 2.92)** |
|  | Total IgG4/ total IgE ratio | 63.31 | 43.75 | 80.71 |  | 0.83 (0.65, 1.05) | 1.04 (0.65, 1.65) |
|  | Total IgE/ cockroach IgE ratio | 2.26 | 5.65 | 19.62 |  | 1.04 (0.76, 1.41) | 1.40 (0.90, 2.18) |
|  | Total IgE/ dust mite IgE ratio | 0.29 | 0.82 | 1.33 |  | 1.03 (0.90, 1.18) | 1.14 (0.95, 1.35) |
| ^†^279 (cytokine responses), 1242 (allergen and *S. mansoni*-specific antibody responses) and 319 (total antibody responses) individuals missing CCA result; not included in analysis  ^‡^Geometric mean ratios and 95% confidence intervals adjusted for the survey design, age and sex.  ^§^SmKK-CCA+ used as the base category  **SmKK-**: Kato-Katz negative result (*S. mansoni*), single stool sample; **SmKK+:** Kato-Katz positive result (*S. mansoni*), single stool sample; **CCA-:** Negative result for *S. mansoni* circulating cathodic antigen; **CCA+:** Positive result for *S. mansoni* circulating cathodic antigen; **SmKK+CCA+/-:** SmKK+ result, irrespective of CCA result; **SWA**: *Schistosoma* worm antigen; **SEA**: *Schistosoma* egg antigen; **aGMR**: adjusted geometric mean ratio; **95% CI**: 95% confidence interval. | | | | | | | |

**References**

1. Hinkle DE, Wiersma W, Jurs SG. Applied Statistics for the Behavioral Sciences 5th Edition: Boston: Houghton Mifflin; 2003.
